# Dietary Strategies in Adult Patients with Eosinophilic Esophagitis: A State-of-the-Art Review

**DOI:** 10.3390/nu15102409

**Published:** 2023-05-22

**Authors:** Carlo Maria Rossi, Marco Vincenzo Lenti, Stefania Merli, Hellas Cena, Antonio Di Sabatino

**Affiliations:** 1 Department of Internal Medicine and Therapeutics, University of Pavia, 27100 Pavia, Italy; 2 First Department of Internal Medicine, Fondazione IRCCS San Matteo, 27100 Pavia, Italy; 3 Laboratory of Dietetics and Clinical Nutrition, Department of Public Health, Experiemental and Forensic Medicine, University of Pavia, 27100 Pavia, Italy; 4 Clinical Nutrition Unit, Department of General Medicine, Istituti Scientifici Maugeri IRCCS, 27100 Pavia, Italy

**Keywords:** allergy, LTP, eosinophils

## Abstract

Allergen-free diets are a specific and effective anti-inflammatory therapy for eosinophilic esophagitis. They should be carried out by a multidisciplinary team to reduce side effects and improve adherence. According to recent guidelines and expert opinions, empirical diets with a reduced number of eliminated food categories and a step-up approach are the most encouraged strategy to reduce the number of endoscopies to identify food trigger(s) and maximize clinical results and adherence. Despite the fact that allergy testing-based diets are not recommended at a population level, geographical sensitization patterns may play a role in some patients in specific areas, such as in Southern and Central Europe.

## 1. Introduction

Eosinophilic esophagitis (EoE) is a chronic, allergen-triggered, immune-mediated disease characterized by eosinophilic infiltration and esophageal dysfunction in the absence of secondary causes. EoE is rapidly on the rise in Western countries, with an incidence of up to 20/100,000 inhabitants per year and a prevalence of up to one case/1000 people. Hence, once considered a rare disease, it currently constitutes a relevant health problem due to the impaired quality of life and overall management costs [1,2,3,4,5].

Genetic and environmental factors play a role in establishing a chronic inflammatory process that shares the cellular and molecular features of other type 2 disorders, such as eczema, allergic rhinitis, asthma and food allergies, but the mechanism underlying its pathogenesis is still elusive.

Clinical symptoms depend on age and disease duration, with dysphagia being the most common complaint and bolus impaction being the most common presentation in adult patients.

Food and environmental factors, among others, are recognized as important triggers of the disease. Of note, the connection between EoE and diet was identified early in the history of this disorder, as attested by the marked clinical-histological improvement of children with EoE through elemental diets consisting of amino acids (allergen-free) and subsequent relapse after food reintroduction. Notably, most of these cases were refractory to acid reflux-targeted therapies and even surgical intervention [6]. Accordingly, diet therapy is a first-line anti-inflammatory treatment for EoE, along with protein-pump inhibitors (PPI) and swallowed topical steroids (STS) [6,7].

The natural history of EoE is characterized by a progressive course leading to fibrosis. Therefore, life-long therapy is usually required. Of note, one distinguishing feature of diet therapy is that it is the only treatment modality that specifically targets the main trigger and cause of the disease. Moreover, most dietary interventions allow the patient to identify the trigger of the disease. Finally, diets allow the long-term management of EoE with a drug-free regimen, which constitutes a significant advantage given the young age of most patients and the chronic nature of the disease.

We herein revise in a narrative fashion the current evidence regarding dietary strategies in adult patients with EoE.

## 2. Methods

The aim of this narrative review is to offer an updated view of dietary interventions in adult patients with EoE to improve the management of these patients through the optimization of dietary therapeutical strategies according to patient characteristics.

In January 2023, we performed a literature search for relevant studies using PubMed. The main medical subject heading terms used were *eosinophilic esophagitis*, diet*, *therapy*, *treatment*, *intervention* and using the qualifier *adult*. Given the peculiar considerations of the pediatric setting, this topic will not be covered in the present review. By using the aforementioned subject headings, we found more than 250 studies. Since inception, we have focused on the original review articles and case reports/series. 

### 2.1. The Positioning of Dietary Therapy in the Management of EoE

According to current guidelines evaluating management strategies for EoE in adults, monotherapy with one of the abovementioned treatments, PPI, STS or diet, is the usual initial approach in the therapeutic algorithm. 

In cases of inadequate response to the first-line treatment, as assessed by a combination of clinical criteria and histological parameters obtained through endoscopy (>15 eosinophils/high power field), one of the other treatments should be started [7,8,9]. Finally, in cases of refractory disease, elemental diets can be exploited as a rescue therapy [7,8,9].

More recently, the joint consensus guidelines issued by the British Society of Gastroenterology and the British Society of Paediatric Gastroenterology, Hepatology, and Nutrition (BSPGHAN) foresee the possibility of combined treatment modalities, i.e., PPI and topical steroids [9]. Other combinations, including dietary interventions such as diet and pharmacological therapy, PPI, or STS, can be considered in non-responsive cases. Moreover, according to these guidelines, a two-food elimination diet (2-FED), including milk and gluten-containing cereals, should be commenced at first before stepping up to more restrictive diets, such as four- or six-food elimination diets (4-FED and 6-FED, respectively), after endoscopic and histologic evaluation between 8 and 12 weeks later [9].

**Figure 1:**
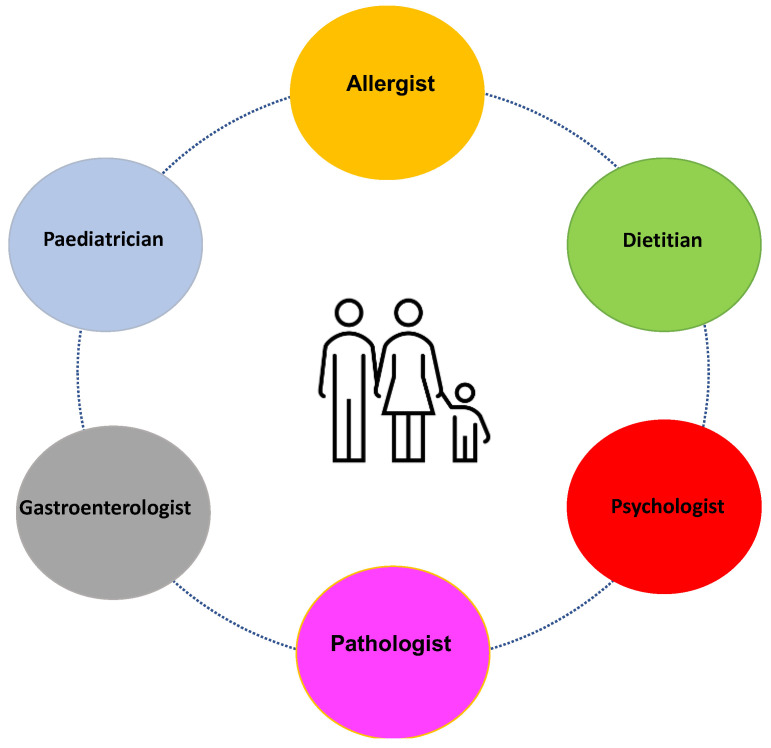
Dietary therapies in eosinophilic oesophagitis should be carried out by a multidisciplinary team comprising, among others, an allergist, a gastroenterologist, a dietitian and a psychologist. The interaction between these professionals is suggested by the circle. The interaction between the allergist for adults and a paediatrician is especially important in the transition age.

Dietary interventions are, however, associated with side effects, including, among others, the risk of malnutrition (especially in milk- and egg-free diets due to a reduced assumption of fat and carbohydrates in wheat- and milk-free diets) and of micronutrient deficiencies (especially in egg-, milk- and wheat-free diets with a reduced assumption of vitamins and calcium), the financial burden on patients and their families, and the risk of the occurrence of new allergic sensitizations to food [10,11,12]. Adherence is also of concern, especially in the long term. Therefore, in line with guidelines indications, to overcome some of these problems, dietary therapy should be supervised by an expert dietitian with allergy competence, an allergist should be included in the care team, psychological support should be offered in case of need, and dietary strategies should be adapted to patient needs, taking into consideration patient age and medical history, feeding behaviours and weight, geographical, social and economic factors, among other elements. The integrated approach to dietary interventions in EoE is described in Figure 1.

### 2.2. Preliminary Considerations for Elimination Diets in the Clinical Setting

The prescription of a dietary strategy should be the result of a shared decision between the doctor and the patient (and their family) and should take into consideration patient features and habits.

Some practical information about the aims and strategies of elimination diets needs to be considered before their use in clinical practice [9,11].

Firstly, the aims of the elimination diets, such as the identification of food triggers and long-term food avoidance of culprit allergens, should be clearly explained to the patients.

The prescription of an elimination diet should be carefully pondered in patients who already have multiple dietary restrictions, such as due to IgE-mediated food allergies or celiac disease, which are present in up to 67% and 25% of patients with EoE, respectively. Similarly, a dietary approach should be avoided in patients with several triggers (usually five) of the disease due to the significant food restrictions required [11].

Finally, in cases of severe symptomatic disease, according to expert opinion, STS should be initially preferred over empiric elimination diets.

Once a dietary strategy is chosen, a step-up approach, starting with the elimination of a reduced number of food categories, usually two in 2-FED, and progressing to 4-FED or 6-FED, the latter in highly motivated patients, is currently the preferred approach to food elimination according to guidelines since it is cost-effective and improves compliance. Specific compliance issues may, however, arise in young adults and adolescents.

To adequately carry out elimination diets, ensuring correct label reading through written information is important. 

**Figure 2:**
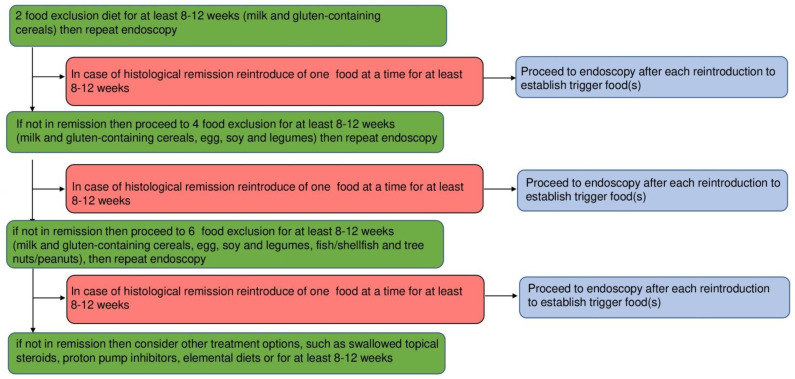
Allergen-free diets have both a diagnostic and therapeutic role. A step-up to food elimination is depicted, from 2 to 6 food and to elemental diets in case of no response. After obtaining histological remission, a single food reintroduction at a time followed by endoscopy is needed to identify the trigger(s). Adapted from [9].

A minimum duration of 6 weeks, usually 8 to 12, for each step of food elimination is usually warranted. After confirming clinical and histological remission through endoscopy, the patient should then reintroduce one food at a time for at least 8–12 weeks and be rescoped after reintroduction to identify the food trigger, as outlined in Figure 2 [9]. Indeed, such a high number of endoscopic examinations within a short time lapse constitutes a relevant burden for patients. Although we do agree with the guidelines, the willingness to undergo follow-up endoscopic examinations should be discussed with patients, with the recommendation of at least the first diagnostic examination and the last follow-up examination at the end of the diet. 

### 2.3. Dietary Treatment Possibilities

**Figure 3:**
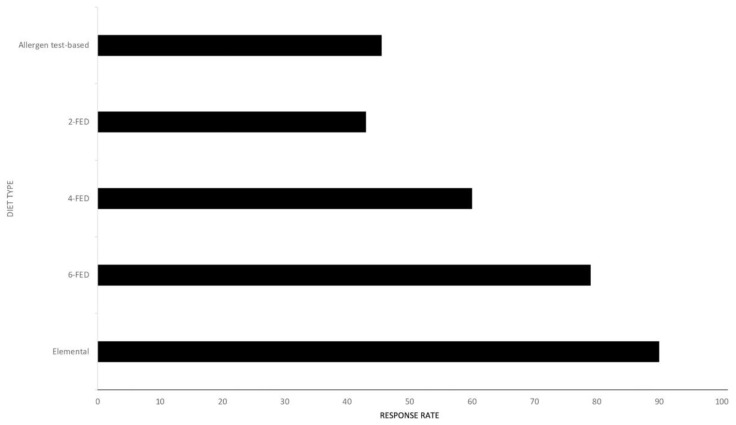
Maximal response rate for the different dietary strategies, including elemental diets, empirical diets and allergen-testing-based ones, according to the available literature on the matter.

There are several widely recognized dietary elimination diets that are associated with varying response rates, as shown in Figure 3. They can prescribe the elimination of some food categories in their entirety and allow feeding through an amino acid formula.

Their design can be based on empirical considerations, thus including the most frequently implied food categories in the general population, such as empirical diets, or on allergy testing performed in the very patient, allergy-based diets, and differ according to the underlying strategies and patient characteristics.

### 2.4. Elemental Diet

The evidence of the complete remission of refractory cases of EoE achieved by feeding for 6 weeks with an amino acid-based or elementary formula (devoid of allergens) constitutes the etiologic demonstration of the allergic nature of EoE [6]. These positive results, initially obtained in children, have been later confirmed in adults. According to guidelines, this strategy may be used as a rescue therapy in both adults and children, and it is particularly suitable for malnourished children in order to avoid further loss of weight [9,10,11]. However, its poor palatability reduces adherence, hampers the acquisition of oral and motor skills in infants, and leads to the development of nutritional deficiencies. Moreover, this strategy does not allow trigger identification [7,8,9,10]. Due to this consideration, elemental diets have a limited role in EoE.

### 2.5. Elimination Diet Based on Food Allergy Testing

Given that allergens trigger EoE in a significant proportion of patients, the use of allergy testing to guide elimination diets has a strong theoretical rationale. Moreover, patients with EoE have frequent allergic comorbidities, including allergic rhinitis and asthma.

However, results of food allergy testing-based diets have been modest in adults and variable in children independently of the test used, such as skin prick tests, specific IgE or recombinant molecules, and patch tests, according to a systematic review and meta-analysis [13]. 

Collectively, the response rate is around 50%. The rate of histologic failure was calculated at 41% in studies in which the patch test was used and 61% in studies in which other tests other than the patch test were used. However, the certainty of this effect estimate was very low due to inconsistencies in the studies included and their single-arm design. It is possible that IgE testing is not accurate in predicting food-related exacerbations since EoE is not an IgE-mediated allergy but a food-induced cell-mediated reaction. Patch tests are performed with the application of food extracts on the skin and have a strong diagnostic rationale since they detect delayed reactions to allergens that are cellular-mediated. However, they lack sensitivity and may not be specific since they do not reflect the local esophageal reaction to food allergens.

Altogether, given the low rate of reproducibility and predictability in trigger identification and the time-consuming procedures of allergic tests in EoE, food allergy testing-based diets are currently not encouraged by most guidelines to choose the type of dietary restriction [9,14]. These limitations and the modest results of food allergy testing-based diets have led to the success of empiric elimination diets.

### 2.6. Empiric Elimination Diets

Empiric elimination diets are based on the elimination of several food/food categories independently of the patient’s sensitization profile/history.

The 6-FED consists of the elimination of the six food groups more commonly associated with the disease, i.e., cow milk or wheat or all gluten-containing cereals, egg, soy, peanut/tree nut, fish, and sea food, as assessed in the paediatric population of Chicago, where the diet was initially devised [15]. Because of cross-reactivity issues, in some cases, all dairy products (comprising goat’s and sheep’s milk) are eliminated together with cow’s milk and all gluten-containing cereals together with wheat.

This diet is highly effective, being associated with a high response rate of up to 75% in children. These results have been later confirmed in prospective studies in adults in both the United States, Europe (Spain) and Australia [16,17,18,19]. A major disadvantage of this dietary strategy is that it requires a relevant number of endoscopies for histological evaluation after each individual food category reintroduction in order to identify the trigger. Moreover, patients should be highly motivated to carry out this diet, which, though less restrictive than an elemental formula, is still demanding. So long-term adherence is an important issue, along with possible associated nutritional deficiencies. For the above considerations, it is more suitable for overweight patients.

Interestingly, in most patients responding to 6-FED, only a few triggers have been identified, usually one or two. This finding constitutes the background for assessing the efficacy of less restrictive, and thus more appealing, dietary strategies. As a result, the 4-FED and the 2-FED have been put forward. Both consist of the elimination of wheat and milk, whereas in the 4-FED, eggs and soy/legumes are also included in the list of foods to be avoided.

A multinational prospective study in 130 patients with EoE, including 25 children, by Molina-Infante (the 2-4-6 study) and colleagues evaluated the effect of a step-up strategy, which consisted of a 2-FED for 6 weeks, gradually followed by a 4-FED and a 6-FED in case of no response [20]. Almost 100 patients took part in all steps of the study.

In responders, at any step, previously eliminated food groups were reintroduced to identify the trigger through endoscopy. The 2-FED strategy achieved histopathology remission in 43% of patients independently of age. Of note, 43% of patients in the study were allowed to receive PPI during 2-FED. 

Milk was the most frequent trigger (52%), along with gluten-containing cereals (16%); both were present in 28% of cases. By stratifying patients by age, milk was identified as the trigger in 33% of children but in a significantly lower percentage of adults (18%).

The remission rates with 4-FED and 6-FED were 60% and 79%, respectively, and, consistent with previous data, most patients (91.6%) had only one or two food triggers. In non-responders to 2-FED and 4-FED, three or more triggers were found.

Compared to a 6-FED diet, the 2-FED in this multistep approach allowed early recognition of two-thirds of patients responding to any empiric diet, whereas the 4-FED identified all patients responding to one or two triggers. 

Altogether, this multistep approach identified the majority of patients responding to a simplified empiric diet early and shortened the diagnostic process by 30% and the number of endoscopies by 20%. Yet some limitations of the 2-4-6 study should be mentioned, among them the absence of a control group, the use of a non-validated symptom scale at that time, and the per-protocol analysis of response to the diet. Moreover, results might not be generalized to other geographical areas with different food habits, namely those not based on staple foods.

In a 1-FED, one food, usually milk or wheat, is eliminated, based on the assumption that milk or wheat represent the most common trigger food in patients with EoE and that up to 70% of patients responding to a 2-FED have one single food trigger. The histological remission with milk elimination is around 68%, according to a meta-analysis [13].

As opposed to children, the elimination of cow’s milk in the 1-FED seems to be associated with a lower rate of histological remission in adults (18–25%), possibly highlighting the diverse importance of this food trigger according to age [20,21]. Moreover, the methodological flaws of the studies carried out in children, such as the inclusion of patients with IgE-mediated allergy to milk treated with milk oral desensitization and of patients with milk immunotherapy-related EoE, seem to tone down the initial encouraging results of this strategy even in children.

Of note, the effectiveness of 1-FED restricting milk is being evaluated in two clinical trials against 6-FED in adults (NCT02778867) and 4-FED in children (NCT02778867).

## 3. Geographical Factors

Empirical diets do not consider local epidemiology, particularly with reference to eating habits and thus sensitization patterns to food allergens.

In Spain, for instance, legumes have been shown to be a particularly frequent trigger of the disease, as opposed to the United States and Australia [14,21]. Legumes appear to be a frequent trigger of the disease in the Mediterranean area as opposed to Central and Northern Europe, according to a paediatric registry study [22].

In the Netherlands, sensitization to the birch pollen allergen, *Bet v 1*, and cross-reactive pathogenesis-related (PR) 10- foods appear frequent (39%) [23].

Moreover, sensitization to some allergens is geographically restricted, such as non-specific lipid transfer protein, nsLTP, which are pan-allergens and are present in the peel of most fruits and vegetables and are a relevant cause of severe IgE-mediated food allergy in the Mediterranean area due to their thermal and proteolytic resistance. 

In a monocentric Italian study of adult patients with eosinophilic disorders of the gastrointestinal tract, including EoE, referred to a third-level center, sensitization/allergy to the peach LTP, *Pru p 3*, was a frequent finding, being present in almost 50% of patients with EoE, frequently with a clinical correlate, ranging from oral cavity-limited symptoms to anaphylaxis. Most patients in this series were already avoiding peaches and other nsLTP-containing foods for IgE-mediated allergies [24]. Peaches are among the food sources with higher concentrations of nsLTP compared to nuts, hazelnuts and wheat.

These findings add further complexity to the management of patients with EoE as far as dietary strategies are concerned since sensitization/allergy to pan-allergens, such as PR-10 or nsLTP, imposes a further dietary restriction on patients with EoE.

In accordance with the concept of geographical sensitization pattern, the finding of cases of EoE induced by sublingual immunotherapy for cedar pollen, which is a frequent cause of respiratory allergic disease in Japan [25].

However, the concept of local allergenic sensitization patterns in EoE has received little attention in therapeutic guidelines/position papers at present.

## 4. Outlook

Despite relevant progress being achieved in the dietary management of EoE, some unmet needs remain, as summarized in Table 1. The main areas of unsolved issues regard the number and categories of eliminated foods, the optimal duration of the elimination diet, the development of new IgE-mediated food allergies, the adoption of less invasive modalities of endoscopic evaluation, such as the Cytosponge, for both the diagnosis of EoE and food reintroduction, and diet long-term efficacy. The current lack of reliable non-invasive biomarkers for EoE is also of importance [11,26,27,28]. 

Despite the consensus about the overt lack of efficacy of allergy-testing-based diets, local epidemiology of sensitization seems to have a role in determining relevant food trigger/allergens (i.e., nsLTP, PR-10) in certain geographical areas, such as Southern and Central Europe.

Future studies should also define which diet treatment, among those available, is the most appropriate for a single patient, which factors predict therapeutic response, and when and which combination therapies are indicated. Moreover, the role of biological therapy with IL-4 and IL-13 for EoE and concomitant IgE-mediated food allergies should be assessed [26,29].

## 5. Conclusions

Dietary strategies represent a relevant therapeutic approach to EoE treatment due to their allergen-specific anti-inflammatory effect. Given the possible development of side effects, allergen-free diets should be carried out by a multidisciplinary team and incorporate patient-centered parameters to increase efficacy and adherence. Future studies should help define therapeutic predictive factors and settings when combination therapies are needed.

## Figures and Tables

**Table 1:** Unmet needs in dietary therapy for EoE.

Variables	Considerations
Factors pertaining to food/food categories to be eliminated	- cross-reactivity (geographic area, avoiding cross-reactive food during a specific aeroallergen season, avoidance of one food or the entire food category, i.e., of all gluten-containing cereals or simply wheat) - local allergenic sensitization pattern
Factors pertaining to food/food categories to be reintroduced	- reintroduction of one food or the entire category (as milk and/or all dairy products) - sequence of reintroduction - allergy consultation in case of suspicion of a (new?) IgE-mediated allergy due to avoidance
Factors pertaining to the patient	- age, gender - need for proton-pump inhibition - concomitant allergic disorders and associated biological therapies (such as anti-IL-4 and/or IL-13 monoclonal antibodies), - local allergenic sensitization pattern - new sensitizations/allergy
Factors pertaining to the diagnostic procedure	- validation of procedures with reduced invasiveness (such as Cytosponge or the String test), - safety of repeated administration of sedating agents/procedures
Factors pertaining to the diet	- long term response/loss of response - duration of the elimination diet - new sensitizations/allergy

Some considerations may apply to more than one variable.

## Data Availability

All data pertaining to the study have been presented in the manuscript. Additional data can be asked of the corresponding author.

## Abbreviations

EoE: eosinophilic esophagitis; FED: food elimination diet; LTP: lipid transfer protein; PR-10: pathogenesis-related-10; STS: swallowed topical steroids.
